# The Future of Giant Cell Arteritis Diagnosis and Management: A Systematic Review of Artificial Intelligence and Predictive Analytics

**DOI:** 10.7759/cureus.75181

**Published:** 2024-12-05

**Authors:** Mohammed Khaleel Almadhoun, Mansi Yadav, Sayed Dawood Shah, Laiba Mushtaq, Mahnoor Farooq, Nsangou Paul Éric, Uzair Farooq, Maryum Zahid, Abdullah Iftikhar

**Affiliations:** 1 Medicine, Al-Bashir Hospital, Amman, JOR; 2 Internal Medicine, Pandit Bhagwat Dayal Sharma Post Graduate Institute of Medical Sciences, Rohtak, IND; 3 Medicine and Surgery, Al-Nafees Medical College and Hospital, Islamabad, PAK; 4 Artifical Intelligence, Foundation for Advancement of Science and Technology (FAST) National University of Computer and Emerging Sciences (NUCES), Lahore, PAK; 5 Artificial Intelligence, New York University, Woodbury, USA; 6 Medicine and Surgery, University of the Mountains, Bangangté, CMR; 7 Artificial Intelligence, Syosset High School, Woodbury, USA; 8 Medicine and Surgery, Lady Reading Hospital, Peshawar, PAK; 9 Medicine, King Edward Medical University, Lahore, PAK

**Keywords:** artificial intelligence, convolutional neural network, deep learning, giant cell arteritis, machine learning, random forest, temporal arteritis

## Abstract

Giant cell arteritis (GCA), a systemic vasculitis affecting large and medium-sized arteries, poses significant diagnostic and management challenges, particularly in preventing irreversible complications like vision loss. Recent advancements in artificial intelligence (AI) technologies, including machine learning (ML) and deep learning (DL), offer promising solutions to enhance diagnostic accuracy and optimize treatment strategies for GCA. This systematic review, conducted according to the PRISMA 2020 guidelines, synthesizes existing literature on AI applications in GCA care, with a focus on diagnostic accuracy, treatment outcomes, and predictive modeling. A comprehensive search of databases (MEDLINE (via PubMed), Scopus, Cochrane Central Register of Controlled Trials (CENTRAL), and Web of Science) from their inception to September 2024 identified 309 studies, with four meeting inclusion criteria. The review highlights the potential of AI to improve diagnostic accuracy through image analysis of color Doppler ultrasound and clinical data, with AI models like random forests, convolutional neural networks, and logistic regression demonstrating effectiveness in predicting GCA diagnosis and relapse after glucocorticoid tapering. Despite these promising findings, challenges such as the need for larger datasets, prospective validation, and addressing ethical concerns remain. The review underscores the transformative potential of AI in GCA care while emphasizing the need for further research to refine and validate AI-driven tools for broader clinical implementation.

## Introduction and background

Giant cell arteritis (GCA), also known as temporal arteritis, is a systemic vasculitis primarily affecting large and medium-sized arteries, with a predilection for the cranial branches of the aorta [1]. This inflammatory condition predominantly affects individuals over 50 years of age, with a higher incidence in women and those of Northern European descent [1,2]. GCA is characterized by granulomatous inflammation of the arterial wall, leading to various clinical manifestations, including headache, scalp tenderness, jaw claudication, and visual disturbances [3]. If left untreated, GCA can result in severe complications, most notably irreversible vision loss [4]. The diagnosis and management of GCA present significant challenges to clinicians due to its diverse clinical presentation and the potential for serious complications. Prompt diagnosis and initiation of treatment are crucial to prevent vision loss and other ischemic complications. However, the current diagnostic approach, which typically involves a combination of clinical assessment, laboratory tests, and temporal artery biopsy, has limitations in terms of sensitivity and specificity [5,6]. Furthermore, the management of GCA requires careful monitoring and individualized treatment strategies to balance the need for effective disease control with the minimization of treatment-related adverse effects [5,6].

In recent years, there has been a growing interest in the application of artificial intelligence (AI) technologies across various medical fields, including rheumatology. AI, encompassing machine learning (ML), deep learning (DL), and computer vision techniques, has shown promise in enhancing diagnostic accuracy, predicting disease outcomes, and optimizing treatment strategies for a range of medical conditions [7]. The potential of AI to analyze complex datasets, identify subtle patterns, and assist in decision-making processes makes it an attractive tool for addressing the challenges associated with GCA diagnosis and management [8,9]. The integration of AI in GCA care could potentially revolutionize several aspects of the disease pathway. In diagnosis, AI algorithms could be developed to analyze clinical data, laboratory results, and imaging studies to improve the accuracy and speed of GCA detection. ML models trained on large datasets of GCA patients could potentially identify novel biomarkers or combinations of clinical features that are predictive of disease presence or severity. Additionally, AI-powered image analysis of vascular imaging modalities, such as ultrasound or magnetic resonance angiography, could enhance the detection of subtle vascular changes indicative of GCA. In the realm of disease management, AI could play a crucial role in predicting treatment response, identifying patients at high risk of relapse, and optimizing glucocorticoid tapering regimens. By analyzing patterns in patient data over time, AI algorithms could potentially assist clinicians in making more informed decisions about treatment escalation or de-escalation [10,11]. Furthermore, AI could contribute to the development of personalized treatment plans by identifying patient subgroups most likely to benefit from specific therapeutic approaches or at higher risk of treatment-related complications [12].

The potential applications of AI in GCA extend beyond direct patient care. In research settings, AI could facilitate the analysis of large-scale genomic and proteomic data to uncover new insights into the pathogenesis of GCA and identify novel therapeutic targets. ML techniques could also be employed to design and optimize clinical trials, potentially leading to more efficient drug development processes for GCA and related vasculitides [10]. Despite the promising potential of AI in GCA care, it is important to acknowledge the challenges and limitations associated with its implementation. These include the need for large, high-quality datasets for algorithm training and validation, concerns about the interpretability and explainability of AI-derived decisions, and the necessity for rigorous clinical validation before widespread adoption. Additionally, ethical considerations surrounding data privacy, algorithmic bias, and the appropriate integration of AI into clinical workflows must be carefully addressed [13].

As the field of AI in medicine continues to evolve rapidly, there is a growing body of literature exploring its applications in various aspects of GCA diagnosis and management. However, to date, there has not been a comprehensive systematic review synthesizing the current evidence and evaluating the potential impact of AI on GCA care. This systematic review aims to address this gap by critically appraising the existing literature on the application of AI in GCA, identifying promising areas of development, and highlighting key challenges and future directions for research. By comprehensively examining the intersection of AI and GCA care, this review seeks to provide clinicians, researchers, and policymakers with a clear understanding of the current state of the field, the potential benefits and limitations of AI applications, and the areas where further research and development are needed. Ultimately, this systematic analysis aims to contribute to the ongoing efforts to improve the diagnosis and management of GCA, potentially leading to better outcomes for patients affected by this challenging condition.

## Review

Materials and methods

This systematic review was conducted to comprehensively evaluate the application of artificial intelligence (AI) in the diagnosis and management of Giant Cell Arteritis (GCA). The review adhered to the PRISMA (Preferred Reporting Items for Systematic Reviews and Meta-Analyses) 2020 guidelines to ensure transparency and rigor in the review process [14].

Search Strategy

A comprehensive literature search was conducted across multiple electronic databases, including MEDLINE (via PubMed), Scopus, Cochrane Central Register of Controlled Trials (CENTRAL), and Web of Science. The search covered all publications from the inception of each database until September 2024. The following search string was employed: "(artificial intelligence OR AI OR machine learning OR deep learning OR neural networks) AND (Giant Cell Arteritis OR Temporal Arteritis OR GCA) AND (diagnosis OR management OR treatment)". Additional relevant studies were identified through manual searching of reference lists from key studies and relevant reviews. No language restrictions were applied during the search.

Eligibility Criteria

Studies were eligible for inclusion if they met specific criteria based on population, intervention, comparison, outcomes, and study design (PICO framework). The population of interest included adult patients (aged 18 years or older) with a confirmed diagnosis of Giant Cell Arteritis (GCA). Studies were included if they focused on the application of AI-based tools or models in the diagnosis or management of GCA, including imaging interpretation, predictive analytics, and decision-support systems. Comparative studies between AI methods and traditional diagnostic or management approaches were also included. The primary outcomes of interest were the diagnostic accuracy (e.g., sensitivity, specificity) of AI tools in identifying GCA and the impact of AI on patient management. Secondary outcomes included time to diagnosis, cost-effectiveness, and clinical outcomes related to treatment. Randomized controlled trials (RCTs), observational studies, cohort studies, and case-control studies were considered for inclusion. Case reports, case series, conference abstracts, grey literature, and non-English language articles were excluded.

Study Selection Process

**Figure 1:**
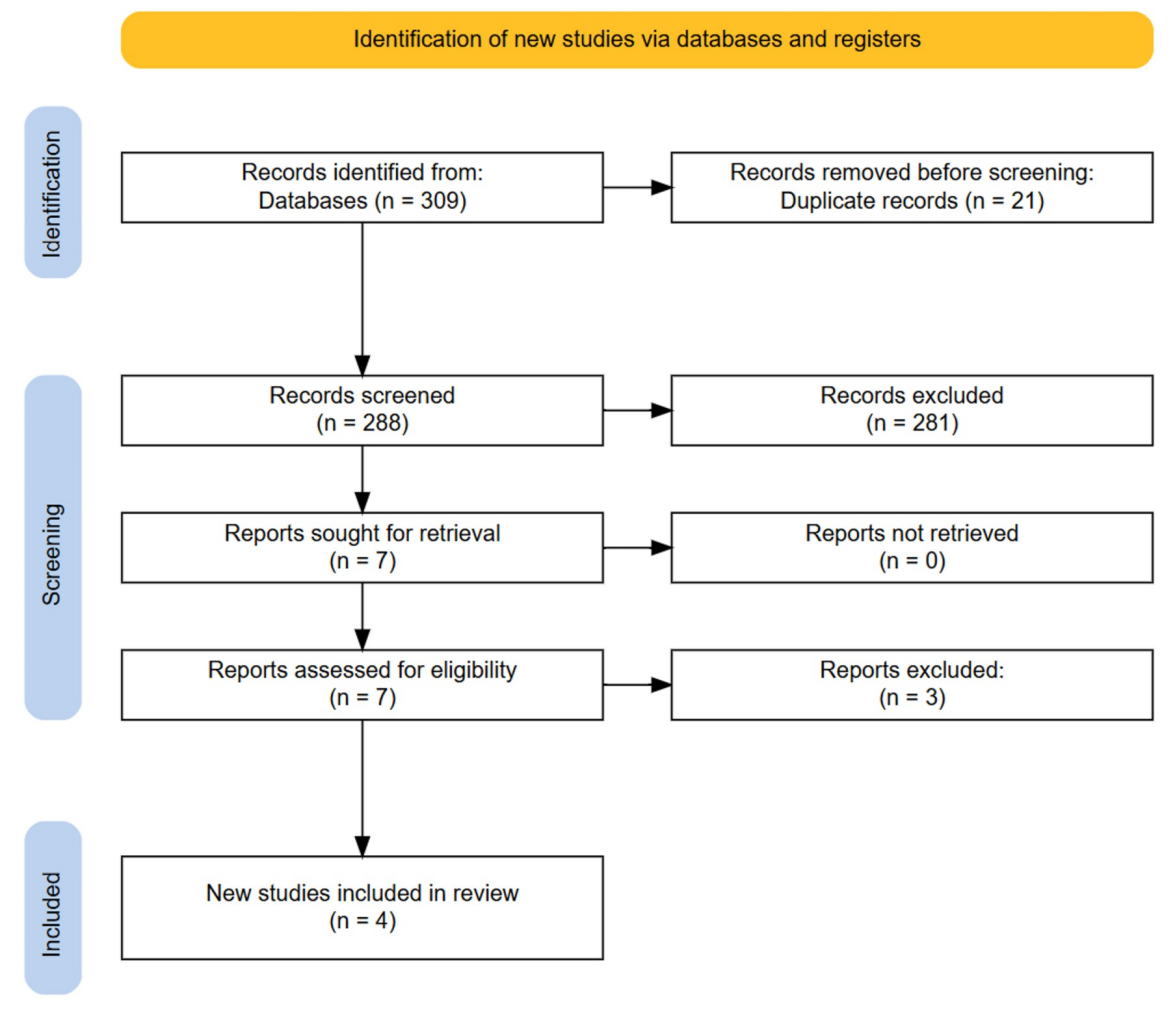
PRISMA diagram showing the study selection process.

Two independent reviewers (S.D. and L.M.) conducted the study selection process. First, titles and abstracts of all identified records were screened to exclude studies that did not meet the inclusion criteria. Full-text articles were then obtained and reviewed by both reviewers to determine final eligibility. Any disagreements regarding study inclusion were resolved through discussion, with a third reviewer (N.P.) consulted if necessary. The study selection process was documented using the PRISMA flow diagram (Figure 1).

Data Extraction

Data extraction was performed using a standardized data extraction form. Two reviewers (U.F and M.Z) independently extracted data from each included study, covering essential aspects such as study design, sample size, patient demographics, AI model details (e.g., type of AI, algorithm, validation method), diagnostic or therapeutic application, and outcome measures (e.g., sensitivity, specificity, accuracy, time to diagnosis, patient outcomes). Any discrepancies in data extraction were resolved through consensus.

Data Analysis

Given the heterogeneous nature of AI applications in GCA, a qualitative synthesis of the findings was conducted. The results from individual studies were summarized and integrated to provide a comprehensive overview of AI's role in improving the diagnosis and management of GCA. Consistencies and discrepancies across studies were highlighted, with a focus on understanding the performance of AI tools in comparison to traditional diagnostic and management methods. Factors influencing variations in outcomes, such as differences in AI models, patient populations, or diagnostic approaches, were also examined.

Results

Study Selection

The initial search identified 309 studies. After removing 21 duplicates, 288 titles and abstracts were screened, of which seven studies were selected for full-text review based on the inclusion criteria. After detailed evaluation, 4 studies met the eligibility criteria and were included in the final systematic review. A manual search of the reference lists did not yield additional eligible studies. The complete study selection process is illustrated in the PRISMA flow diagram (Figure 1).

Study Characteristics

The studies included in this systematic review highlight various applications of AI in the diagnosis and management of GCA. The research explored the use of different AI models to address the challenges of diagnosing GCA and managing patient treatment, focusing on enhancing diagnostic accuracy, reducing delays in diagnosis, and optimizing treatment regimens. The studies reviewed were retrospective cohort studies, reflecting the ongoing efforts to apply ML models to clinical datasets. These retrospective studies aimed to validate AI models using previously collected patient data. The focus on retrospective designs highlights the exploratory nature of AI application in GCA, as real-time prospective data collection and implementation of AI in clinical settings remain limited. However, the studies demonstrate the potential of AI to analyze existing clinical data and predict outcomes effectively.

The sample sizes across the studies varied significantly. For instance, Venerito et al. (2022) included 107 patients with classified GCA, while Roncato et al. (2020) worked with a dataset containing 1,311 color Doppler ultrasound (CDU) images from 137 patients [15,16]. The studies by Ing et al. analyzed data from 1,201 and 530 participants [17,18]. The variation in sample sizes reflects the heterogeneity in data availability, particularly regarding imaging datasets, which often require large numbers of high-quality images for effective AI model training. Additionally, the demographic characteristics of patients varied slightly between studies. The mean age of participants in most studies ranged from 70 to 75 years, consistent with the typical age distribution of GCA, which predominantly affects individuals over 50. Women represented the majority of the study populations, reflecting the higher incidence of GCA in females. A variety of AI models were used across the studies, showcasing the diversity of ML approaches applied to GCA. The diversity in AI models reflects the exploratory nature of AI in GCA diagnosis and management. ML models, such as random forests, logistic regression, and neural networks, are suitable for handling structured clinical data, while CNNs are more adept at processing complex imaging data.

The studies targeted two primary applications of AI in GCA care: diagnosis and management. In the diagnostic realm, AI models were used to assist in predicting GCA based on clinical and imaging data. For instance, Roncato et al. (2020) focused on the automated detection of the halo sign, a key feature in GCA diagnosis, using CDU images [16]. This approach showed high specificity, demonstrating the potential of AI in augmenting diagnostic imaging interpretation. On the management side, Venerito et al. (2022) explored the use of AI to predict disease flare after glucocorticoid tapering [15]. By identifying patients at higher risk of relapse, AI can support clinicians in making informed decisions about adjusting treatment plans and preventing complications.

The AI models demonstrated promising results, particularly in enhancing diagnostic accuracy and predicting treatment outcomes. For example, Venerito et al. (2022) reported that their random forest model accurately predicted GCA relapse after glucocorticoid tapering with an accuracy of 71.4% [15]. This predictive capability can assist clinicians in personalizing treatment plans and potentially reduce the risk of relapse. Similarly, Roncato et al. (2020) achieved high specificity (97.1%) with their CNN model in detecting the halo sign on CDU images, suggesting that AI-powered image analysis could be used to improve the reliability of GCA diagnosis [16]. In comparison, Ing et al. found that both neural networks and logistic regression models could accurately predict GCA diagnosis based on clinical data, with AUROC values around 0.86 [17,18]. However, the false-negative rates in these models remain a concern, highlighting the need for further refinement to reduce diagnostic errors. The summary of the findings of the included studies is mentioned in the following table (Table 1).

**Table 1:** Summary of key findings from included studies. GCA: Giant Cell Arteritis, AI: Artificial Intelligence, ML: Machine Learning, DL: Deep Learning, RF: Random Forest, CNN: Convolutional Neural Network, LR: Logistic Regression, CDU: Color Doppler Ultrasound, TAB: Temporal Artery Biopsy, ESR: Erythrocyte Sedimentation Rate, AP: Average Precision, MCR: Misclassification Rate, FN: False Negatives, AUROC: Area Under the Receiver Operating Characteristic Curve

Study	Year	Journal	Country	Study Design	Sample Size	Age Range	Gender	AI Model Used	Training Data Size	Validation Method	Diagnostic or Therapeutic Focus	Results	Conclusions
Venerito et al. [15]	2022	Frontiers in Immunology	Multi-centre Study	Retrospective cohort study	107 patients with classified GCA	Mean age 74.1 ± 8.5 years	73 women (68.2%), 34 men (31.8%)	RF, LR, DT	Not explicitly stated	5-fold cross-validation	Predicting GCA flare after glucocorticoids tapering	RF showed best performance: accuracy 71.4%, precision 72.1%, recall 60%, AUROC 0.76. LR: accuracy 70.4%, precision 62.6%, recall 62.5%, AUROC 0.73. DT: accuracy 62.9%, precision 50.8%, recall 47.5%, AUROC 0.65	RF algorithm can predict GCA relapse after glucocorticoids tapering with sufficient accuracy. The most important predictive factors were high ESR at baseline, comorbid diabetes, and presence of polymyalgia rheumatica. This ML method represents a reproducible tool capable of supporting clinicians in GCA patient management. External validation in independent cohorts is needed
Roncato et al. [16]	2020	Clinical and Experimental Rheumatology	Multi-centre Study	Retrospective cohort study	137 patients (1,311 CDU images)	Mean age 73.6 ± 8.9 years (Group 1), 72.6 ± 12.1 years (Group 2)	Not explicitly stated for overall sample	CNN with U-Net architecture	627 images from 65 patients	Separate validation (342 images from 38 patients) and test (342 images from 34 patients) sets	Diagnostic - automated detection of halo sign on color Doppler ultrasound (CDU) images for giant cell arteritis	AUC of 0.931 and 0.835 for validation and test sets respectively. Using 1,200 pixel threshold: Validation set: Sensitivity 68.8%, Specificity 97.1% Test set: Sensitivity 60.0%, Specificity 95.3%	The DL model showed promising results in automated detection of halo sign on CDU images for GCA diagnosis, especially on standardized images. Performance could be improved with larger datasets and image standardization. This tool could potentially aid diagnosis, staff training and student education.
Ing et al. [17]	2019	Clinical Ophthalmology	Multi-centre Study	Retrospective cohort study	1,833 (1,201 with complete data)	38-98 years	68.7% female in negative biopsy group, 71.2% female in positive biopsy group	ANN and LR	Not explicitly stated	Internal validation using tenfold cross-validation and external validation	Diagnostic prediction	Area under ROC curve: LR 0.867 (95% CI, 0.794, 0.917), NN 0.860 (95% CI, 0.786, 0.911). Misclassification rate: LR 20.6%, NN 18.1%. False-negative rate: LR 47.5%, NN 30.5%	Statistical models can aid in the triage of patients with suspected GCA. Misclassification remains a concern, but cutoff values for 95% and 99% sensitivities are provided.
Ing et al. [18]	2019	Canadian Journal of Ophthalmology	Canada	Comparison study	530	Not specified	Not specified	SVM and LR	280 (210 negative, 70 positive TABx)	Test set of 250 (187 negative, 63 positive TABx)	Diagnostic (predicting temporal artery biopsy outcomes)	SVM: AUC 0.825, AP 0.670, MCR 0.168, FN 0.571; Logistic Regression: AUC 0.827, AP 0.676, MCR 0.184, FN 0.524	SVM did not offer any distinct advantage over logistic regression prediction model in this dataset

Quality assessment

The methodological quality of the included studies was evaluated using the Joanna Briggs Institute (JBI) critical appraisal tools for cohort and case-control studies (Table 2). Based on the JBI critical appraisal tool assessment of the four included studies, two studies (50%) were classified as high quality: Venerito et al. (2022) and Roncato et al. (2020) [15,16]. Both demonstrated robust methodological approaches with clear study parameters, valid condition identification methods, and appropriate statistical analyses, though Roncato et al. had minor limitations in follow-up documentation. The remaining two studies (50%) by Ing et al. were assessed as moderate quality [17,18]. While these studies maintained appropriate statistical approaches and valid outcome measurements, they showed limitations in follow-up documentation and confounding factor management. This quality assessment suggests that while the current evidence base includes methodologically sound studies, there is room for improvement in future research, particularly in areas of follow-up documentation and handling of confounding factors.

**Table 2:** Quality assessment of the included studies using the Joanna Briggs Institute (JBI) critical appraisal checklist.

JBI Critical Appraisal Criteria	1. Were the two groups comparable other than the presence of disease in exposed group?	2. Were the exposures measured similarly to create comparable groups?	3. Was the exposure measured in a valid and reliable way?	4. Were confounding factors identified?	5. Were strategies to deal with confounding factors stated?	6. Were the groups/participants free of the outcome at the start of the study?	7. Were the outcomes measured in a valid and reliable way?	8. Was the follow-up time reported and sufficient to be meaningful?	9. Was follow-up complete, and if not, were the differences between groups statistically analyzed?	10. Was the statistical analysis appropriate?	Total Score (out of 10)	Quality Level
Venerito et al. [15]	Yes	Yes	Yes	Yes	Yes	Yes	Yes	Yes	Yes	Yes	10	High
Roncato et al. [16]	Yes	Yes	Yes	Partially	Partially	Yes	Yes	Partially	Partially	Yes	8	High
Ing et al. [17]	Partially	Partially	Yes	Partially	No	Partially	Yes	No	No	Yes	6	Moderate
Ing et al. [18]	No	Partially	Partially	No	No	Partially	Yes	No	No	Yes	5	Moderate

Discussion

The pathophysiology of GCA involves a cascade of immune-mediated events leading to granulomatous inflammation of the arterial wall. This process is characterized by the infiltration of T-cells, macrophages, and multinucleated giant cells, which contribute to intimal hyperplasia and luminal occlusion. The exact trigger for this inflammatory response remains unclear, but it is believed to involve a combination of genetic predisposition and environmental factors [1,19,20]. The diagnosis of GCA traditionally relies on a combination of clinical assessment, laboratory tests, and histopathological examination. Elevated inflammatory markers, particularly erythrocyte sedimentation rate (ESR) and C-reactive protein (CRP) are characteristic but not specific to GCA [1]. Temporal artery biopsy (TAB) has long been considered the gold standard for diagnosis, offering high specificity. However, the sensitivity of TAB is limited due to the potential for skip lesions and the impact of prior glucocorticoid treatment. Moreover, the invasive nature of the biopsy and the expertise required for proper interpretation can lead to delays in diagnosis and treatment initiation [21,22]. In recent years, imaging modalities have gained prominence in the diagnostic algorithm for GCA. Color Doppler ultrasonography (CDUS) of the temporal arteries has emerged as a valuable non-invasive tool, with the "halo sign" being a characteristic finding indicative of vessel wall inflammation. High-resolution magnetic resonance imaging (MRI) and computed tomography angiography (CTA) offer additional insights into vessel wall changes and can help assess large-vessel involvement. However, the interpretation of these imaging studies requires expertise and can be subject to inter-observer variability [23].

The treatment of GCA centers on the prompt initiation of high-dose glucocorticoids to suppress inflammation and prevent ischemic complications. While effective, long-term glucocorticoid therapy is associated with significant adverse effects, necessitating careful monitoring and individualized tapering strategies. The introduction of tocilizumab, an interleukin-6 receptor inhibitor, has marked a significant advancement in GCA management, offering a steroid-sparing option for patients with refractory or relapsing disease. Ongoing research into targeted therapies aims to further refine treatment approaches and minimize glucocorticoid exposure [24]. The complexity of GCA diagnosis and management, coupled with the potential for severe complications, has led to increasing interest in the application of AI and ML in this field. Our systematic review has identified several key areas where AI technologies are being explored to enhance GCA care.

In the realm of diagnosis, AI models have shown promise in analyzing clinical data to predict the likelihood of GCA. The studies by Ing et al. (2019) demonstrated that both neural networks and logistic regression models could accurately predict GCA diagnosis based on clinical parameters, with the area under the receiver operating characteristic curve (AUROC) values around 0.86 [17]. This suggests that AI-powered decision support tools could potentially assist clinicians in triaging patients with suspected GCA, particularly in settings where specialist expertise may be limited. Image analysis is another area where AI shows significant potential. The work by Roncato et al. (2020) on automated detection of the halo sign in color Doppler ultrasound images is particularly noteworthy. Their convolutional neural network achieved high specificity (97.1%) in identifying the halo sign, a key diagnostic feature of GCA [16]. This application of AI could potentially standardize and expedite the interpretation of vascular imaging studies, reducing inter-observer variability and improving diagnostic accuracy. In the domain of disease management, AI models are being developed to predict treatment outcomes and optimize therapeutic strategies. Venerito et al. (2022) explored the use of ML algorithms to predict GCA relapse after glucocorticoid tapering. Their random forest model demonstrated an accuracy of 71.4% in predicting disease flare, highlighting the potential of AI in supporting personalized treatment decisions [15]. By identifying patients at higher risk of relapse, such models could guide more tailored glucocorticoid tapering regimens and inform the timely initiation of steroid-sparing therapies.

Limitations and future directions

This systematic review has several limitations that should be considered when interpreting its findings. First, the small number of eligible studies (n=4) reflects the nascent state of AI applications in GCA, limiting the generalizability of conclusions. The studies included were predominantly retrospective in nature, lacking prospective validation in diverse clinical settings. Sample sizes varied considerably across studies, with some utilizing relatively small datasets that may not fully capture the heterogeneity of GCA presentations. The AI models employed across studies were heterogeneous, making direct comparisons challenging. Additionally, most studies focused on specific aspects of GCA care (e.g., diagnosis or relapse prediction) rather than providing a comprehensive AI-driven approach to GCA management. The lack of standardization in outcome measures and validation methods further complicates the synthesis of results. Importantly, the studies reviewed did not extensively address the interpretability and explainability of AI models, which is crucial for clinical adoption. The potential for bias in AI algorithms, particularly related to demographic factors, was not thoroughly explored. Furthermore, the studies did not adequately assess the integration of AI tools into existing clinical workflows or evaluate their impact on patient outcomes and healthcare resource utilization. Ethical considerations, including data privacy and the appropriate balance between AI-assisted and human decision-making, were not comprehensively addressed. Lastly, the rapid evolution of AI technologies means that some of the models and techniques described may already be outdated, highlighting the need for ongoing research and updating of AI applications in GCA care.

Looking to the future, several promising directions emerge for AI applications in GCA. The development of multimodal AI models that integrate clinical, laboratory, and imaging data could potentially offer more comprehensive and accurate diagnostic and prognostic predictions. Such models could assist in early detection of GCA, potentially before the onset of irreversible complications. In the realm of treatment, AI could play a crucial role in optimizing glucocorticoid dosing and tapering regimens. By analyzing patterns of disease activity, biomarker levels, and patient-specific factors, AI models could potentially predict individual responses to treatment and guide more personalized management strategies. This could help strike a balance between effective disease control and minimization of treatment-related adverse effects. Another exciting prospect is the application of AI in drug discovery and development for GCA. ML techniques could be employed to analyze large-scale genomic and proteomic data, potentially uncovering new therapeutic targets and accelerating the development of novel targeted therapies. The role of AI in facilitating telemedicine and remote monitoring for GCA patients is also an area ripe for exploration. AI-powered systems could potentially assist in the remote assessment of disease activity, enabling more timely interventions and reducing the need for frequent in-person visits.

## Conclusions

The application of artificial intelligence in the diagnosis and management of giant cell arteritis represents a promising frontier in rheumatology. While the current body of evidence suggests potential benefits in enhancing diagnostic accuracy, predicting disease outcomes, and optimizing treatment strategies, significant work remains to be done to translate these early findings into clinically validated tools. As artificial intelligence technologies continue to evolve and larger datasets become available, we anticipate a growing integration of artificial intelligence-assisted decision support in the care of giant cell arteritis. However, it is crucial that this integration occurs in a thoughtful and evidence-based manner, with careful consideration of the ethical, practical, and clinical implications. The ultimate goal should be to leverage artificial intelligence as a powerful adjunct to clinical expertise, enhancing our ability to provide timely, accurate, and personalized care for patients with giant cell arteritis.
